# "I won't be staying here for long": a qualitative study on the retention of migrant nurses in Ireland

**DOI:** 10.1186/1478-4491-7-68

**Published:** 2009-08-06

**Authors:** Niamh Humphries, Ruairi Brugha, Hannah McGee

**Affiliations:** 1 Division of Population Health Sciences, Royal College of Surgeons in Ireland, Dublin, Ireland

## Abstract

**Background:**

Although international nurse recruitment campaigns have succeeded in attracting large numbers of migrant nurses to countries such as Ireland, where domestic supply has not kept pace with demand, the long-term success of such initiatives from a workforce planning perspective will depend on the extent to which these nurses can be retained in destination countries.

**Methods:**

This paper draws on qualitative, in-depth interviews undertaken with 21 migrant nurses in Ireland, focusing specifically on their future migration intentions.

**Results:**

Our findings indicate that more than half of the respondents are considering migration onwards, for the most part because the destination country has failed to provide them with sufficient stability, particularly in terms of citizenship and family reunification. In considering onward migration, factors outside the health system were of most concern to those interviewed.

**Conclusion:**

This demonstrates the need for destination countries to take a broader and more long-term approach to international nurse recruitment, rather than regarding it as an inexpensive way to fill gaps within the health care system.

## Background

### The need to retain as well as recruit

Active overseas recruitment strategies have succeeded in attracting large numbers of migrant nurses to countries where domestic production and retention have not kept pace with growing needs [1,2]. However, it is destination countries' ability to retain these nurses that will determine the long-term effectiveness of these workforce strategies. Research has shown that attention to factors promoting retention is important to the long-term success of active international recruitment campaigns, which otherwise will have minimal impact beyond the short-term relief of staff shortages [3-8]. The Irish experience of international recruitment and retention, as presented in this paper, is a prime example of a country heavily reliant upon migrant health workers. As such, the lessons learnt may have a wider applicability internationally.

Migrant nurses play a significant role in the Irish health system – 40% of all nurses newly registered in Ireland between 2000 and March 2009 were from outside the European Union (Irish Nursing Board, unpublished data). Some hospitals in the Irish capital have identified 50% to 80% of their nursing staff as migrant [9]. The need for migrant nurses in this system is likely to continue for the foreseeable future, a fact borne out by the fact that many migrant nurses hold permanent jobs within the health system and also by the recent statement from the Manager of the National Recruitment Service that: "There shall continue to be an ongoing need for international nurses mainly in specialist areas" [10].

Health employers often rely on anecdotal evidence to reassure them of the stability and long-term intentions of their migrant nurse workforce. For instance, the major State health employer in Ireland recently claimed to have no evidence to suggest that migrant nurses employed by them intend to leave Ireland [9]. In his research with health care managers in the United Kingdom, Buchan unearthed a similar and untested assumption, i.e. most believed that their migrant nurse employees would remain in the United Kingdom and cited family reunification and United Kingdom house purchases as evidence of that stability [5]. However, a recent survey of migrant nurses in the United Kingdom appeared to contradict the assumption of stability. Of those migrant nurses surveyed, just under half (43%) were considering a move to another country, with one third (32%) having been contacted by recruitment agencies and offered work outside the United Kingdom in the previous six months [11].

The present study – the Nurse Migration Project – sought to consult with migrant nurses to obtain evidence, through qualitative and quantitative research methods, to assist employers and policy-makers in making informed decisions regarding their migrant nurse workforce. Given the extent to which Ireland has come to rely on migrant nurses [2], this is of fundamental importance to the Irish health system. This paper presents qualitative research findings from the Nurse Migration Project, focusing specifically on the factors likely to influence migrant nurses' decisions to remain in Ireland or migrate onwards, but the findings speak to an international audience and serve as a timely reminder of the experiences of individuals caught up in the "strange version of musical chairs" [4] that is international nurse recruitment.

### Global context

The struggle to retain migrant nurses in Ireland takes place, as their recruitment did, in the context of an "international war for skills" [4]. Developed countries compete with each other to recruit from the same global pool of nurses. Migrant nurses with overseas experience in an English-speaking country are highly sought-after. This inevitably means that developed countries compete with each other to attract nurses, as Buchan explains:

"The UK has become very reliant on recruiting internationally in the past few years; it can have no complaints when other countries make sure of the same 'solution' to shortages – even if it becomes a target as well as a destination" [12].

Canadian employers and recruitment agencies have been actively recruiting nurses from Ireland, using the lure of "affordable housing ... and no rush-hour traffic" [13]. This is a tempting offer for nurses faced with the high rent or house purchase costs and significant daily commutes in Ireland.

While statistics on nurse emigration are incomplete, there are indications that at least some of Ireland's migrant nurses are considering such a move [2]. This is borne out in recent statistics from the Irish Nursing Board which in 2008 saw 3108 verification requests lodged, 69% of which related to nurses from India and the Philippines (Irish Nursing Board, unpublished data) [14]. In 2007, there were 1140 verification requests to the Irish Nursing Board, 45% of which came from Indian and Filipino nurses (Irish Nursing Board, unpublished data)[14].

Although not a precise measure of nurse emigration, verification requests – the procedure through which national nursing boards verify the Irish registration of a nurse seeking to register in their country – are generally considered an indication of intent to migrate. It would appear that the global "carousel" [15] continues, as some of the migrant nurses whom Ireland actively recruited are recruited once again, this time by Australia, Canada or the United States of America.

The OECD notes that, despite the global shortage of nurses, "most countries do not have specific retention policies for foreign health workers, even when the latter represent a large share of the workforce" [16]. Perhaps destination countries find it cheaper to continue to recruit internationally rather than to instigate the change – to policy and practice – required to retain migrant nurses in post [17]. However, this approach is unsustainable in the long term and also suggests a disregard for the impact of onward migration on the lives of individual migrant nurses and their families.

## Methods

Ethical approval for the study was granted by the Research Ethics Committee of the Royal College of Surgeons in Ireland and, in 2007, in-depth interviews were conducted with 21 migrant nurses working in Ireland. In recruiting a sample, the researchers sought to ensure that the sample was heterogeneous – including migrant nurses from a range of countries; varying in age, marital status and duration of working in Ireland; active and passive recruits; and those working in both the public and private sectors.

Unfortunately, beyond data on nationality and year of arrival as derived from immigration and registration data, no further information was available on the overall migrant nurse population in Ireland. Thus only limited estimates of generalizability between the sample and Ireland's migrant nurse population can be made.

Drawing on available immigration data, 45% of visas issued to migrant nurses between 2000 and 2008 went to nurses from India; a further 45% went to nurses from the Philippines, with much smaller numbers (2%) issued to nurses from Australia, Nigeria and South Africa and the remaining 3% of visas issued to nurses from 42 other countries (Irish Department of Enterprise, Trade and Employment, unpublished data). Immigration data also indicate that 35% of migrant nurse visas were issued between 2000 and 2002, 14% in 2003–2004, 35% in 2005–2006 and 16% in 2007–2008 (Irish Department of Enterprise, Trade and Employment, unpublished data). Insofar as possible, the researchers sought a sample of migrant nurses that would correspond to the overall population of migrant nurses in Ireland.

Gaining access to a sample of migrant nurses proved difficult. Initially, potential respondents were contacted via the Overseas Nurses Section of the main nursing union, the Irish Nurses Organisation. This approach yielded a low response: from 250 randomly selected migrant nurses to whom letters were forwarded on behalf of the research team, only eight responses were received. Similar disappointing responses have been recorded by researchers in the United Kingdom who sought to contact migrant nurse respondents by post via the Royal College of Nursing [18,19].

We can only speculate as to the reason for the low response rate – postal addresses may have changed, perhaps those contacted were reluctant to participate in face-to-face interviews, had more pressing demands on their time or simply had little interest in the research topic. The low response meant that alternative recruitment strategies were pursued – articles were placed in migrant newspapers and snowball sampling was also employed – a process of chain referral whereby respondents and gatekeepers are used to refer the researcher to other potential respondents [20].

Limitations of the sample include its small size and its overrepresentation of earlier arrivals and Filipino nurses. The small sample size initially came about as a result of recruitment difficulties, but once interviewing began, it soon became clear that the "rich and experiential"[21] data emerging from the interview necessitated a small sample size to ensure that the quantity of data remained at manageable levels. A point of data saturation was quickly reached – the point at which the researcher felt that increasing the number of respondents would provide no further insights into the research topic, but would result only in "a more cumbersome dataset" [22].

The sample of migrant nurses comprised 16 nurses from the Philippines, four from India and one from Nigeria. The overrepresentation of Filipino respondents is explained by the existence of strong community bonds within the Filipino community in Ireland, which facilitated the success of snowball sampling.

Respondents were predominantly women, with only two men participating in the research. On arrival in Ireland, eight respondents were aged in their 40 s, eight in their 30 s and five were in their 20 s. Most (15) of the respondents were married, three were single, two were divorced or separated and one was widowed. In addition, most (17) respondents had children or were expecting a child at the time of interview.

Fifteen of those interviewed were based in Dublin, while a further six were based outside the capital. Eleven respondents had arrived in Ireland in 2000–2001; three arrived in 2002–2003 and seven arrived in 2004–2005.

Fourteen had been recruited to work in hospitals, four to private nursing homes and another three to work in disability services (operated by charitable organizations). Eighteen respondents had been actively recruited to Ireland, one had emigrated to join a spouse and two others had emigrated independently.

Interviews were conducted in non-workplace settings, as it was felt that this would facilitate a free and open discussion of experiences by respondents. The researcher (lead author) conducted 16 of the interviews in respondents' homes, which provided a familiar setting in which respondents would feel comfortable discussing their experiences [23], and conducted the remaining interviews, at respondents' request, in the research institution. Interviews lasted an average of 69 minutes.

Each interview began with a discussion of confidentiality during which respondents were invited to select a pseudonym to ensure the anonymity of their responses in various research outputs. Interviews progressed to cover topics such as the decision to migrate, the recruitment process, orientation and adaptation programmes, nursing and living in Ireland and future plans. It concluded with a brief discussion of topics the researcher considered to be more "sensitive", such as, for example, remittances and the ethical issues raised by overseas nurse recruitment. On completion of the interview, all respondents were presented with a modest gift voucher to thank them for their participation and to cover any costs incurred [24].

Interviews were audio recorded and were transcribed verbatim. Data analysis was undertaken on an ongoing basis throughout the data collection phase [22], as the researcher familiarized herself with emerging research themes. A general inductive analysis was undertaken via a thorough re-reading of interview transcripts [25], which enabled the researcher to identify emerging key issues, concepts and themes. "Inductive approaches ... aid an understanding of meaning in complex data through the development of summary themes or categories from the raw data" [26]. Data management was facilitated by the use of the MaxQDA qualitative data analysis package.

## Results

Of the 21 nurses interviewed, only four stated that they intended to remain in Ireland on a long-term basis. Over half (11) of those interviewed expressed their intention to emigrate from Ireland within five years – three respondents had made definite plans to emigrate to Canada and a further eight intended to leave Ireland within five years – some to return home, some to migrate to the United States or Australia. Six respondents felt that they would probably stay in Ireland, but qualified this decision either with a discussion of the opportunities available overseas, particularly in the United States, or by stating that their decision to remain was dependent on the employment and migration status of their spouses and children. The following discussion offers an insight into the complex web of factors considered by respondents in deciding whether to stay or to leave.

### Reasons to remain in Ireland

Most of the 10 respondents who planned to remain in Ireland for the foreseeable future sought to do so because they felt that they and their families had settled and also because they wished to avoid the disruption entailed by onward migration:

"It's so traumatic for kids, like, to adapt again, they don't want. I want, but they said, no, no, mam, we're not going, no, we have our friends and we left our friends there and we have now our friends here in Ireland and then we'd be leaving them again" (Agatha, Philippines, 50 s).

Career-related issues, such as the availability of salaries sufficient to enable remittances to family back home, job security or permanency, maternity benefits and educational opportunities were also cited as reasons for staying in Ireland. However, direct financial issues played a less significant role than had been anticipated and were found to be less likely to feature as deciding factors in the decision-making process. Two respondents who stated that they would probably remain in Ireland felt that it compared favourably to other potential migration destinations. For these comparisons, they drew on their own experiences of nursing in the Middle East and their friends' experiences in the United States:

"I went to New York, I went to Missouri, I went to New Jersey, but I've seen the pace of life is different, as compared to here. Like [there] it's all work, work, work, work, work, work, work, work for them and they got home, you know, tired and they leave and they go to another job" (Helmie, Philippines, 40 s).

“Like if I have to look back now to my classmates back home who’re still there back in the Philippines still applying for this kind of job, or they’re still back in Saudi Arabia(...), well I could say, ‘thank God I’m here, thank God I’m in this place where I feel safe” (Fatima, Philippines, 30s).

The desire to be settled, as expressed by respondents, contrasts with the stereotype of the migrant nurse as an extremely mobile individual, constantly seeking better opportunities internationally [18,27]. Although respondents did, to an extent, "rank" destination countries, in doing so they considered a broad range of quality-of-life issues far beyond straightforward salary comparisons.

### The reality of migration

Despite these 10 respondents' having elected to remain in Ireland for the foreseeable future, they were frank about the difficulties inherent in living and working as a migrant nurse in Ireland. Most had made personal sacrifices:

"I've really thought, sometimes I thought, like is it worth coming and working in Ireland? ... In my own country, if I have been in my own country, I would have been a lecturer now, I'd have been worked in a college of nursing, I would have done that and would have done this, I would have had more responsibility" (Sheela, India, 20 s).

Although there are financial benefits to be obtained through migration, in that salary levels in destination countries exceed those available in source countries, even health workers with considerable teaching/management experience found their employment opportunities restricted to frontline nursing care. Padarath identifies this situation as "brain wastage", whereby "highly trained health personnel have been expected to carry out basic, menial tasks" [28]. Such deskilling is neither in the interest of individual migrant nurses nor in the interest of the health systems in which they work.

Respondents found that the high cost of living in Ireland also diminished salary values and reduced the amount that they were able to remit:

"When you're here, like, you want to help your family as well, like, your cousins, your relatives, send money for them, but if you're not able to do that, like, the satisfaction is less" (Sheela, India, 20 s).

Others noted that although working in Ireland was financially attractive, remaining meant living apart from friends and family:

"Yeah, it's not so easy, leaving your friends, your family. Yeah, you have everything here, we can buy everything, we can buy our house, our car there, but Filipino family are not just looking for money, for financial, but for stability as well" (Clara, Philippines, 30 s).

Family featured as an important consideration for migrant nurses in the decision-making process; this was true for both married and single respondents. If they lived apart from family members, the focus was on maintaining the remittance flow to them and on holiday entitlements that would allow family unity, even for a brief period. The social strain of migration in terms of family separation [4] was apparent in respondents' testimonies. For those respondents who lived with family members, the desire for stability and continued family unity was central to the decision to stay or leave.

Overall, respondents were frustrated that they and their families received no entitlements to residency or citizenship as a family unit in return for their service to the Irish health system. Although most held permanent posts within the health system and that provided them with stability of employment, this was not matched in terms of the availability of a long-term, secure immigration status for migrant nurses and their families. This was a concern expressed by single nurses as well as those who were married or who had children:

"At the moment, I'm single, I'm okay, but I'm still, I know, I'm sure in the future soon, I'll get married have my family" (Clara, Philippines, 30 s).

### Reasons to leave Ireland

#### Stability and integrity of the family unit

The desire both for stability and to maintain the integrity of the family unit played a significant role in respondents' decisions to leave Ireland. Of the three respondents who had made definite plans to emigrate, two were doing so in order to ensure that they and their adult children (i.e. aged over 18) could live together as a family unit. The third respondent with definite emigration plans was emigrating as a direct result of the pre-2004 restrictions on work entitlements for dependent spouses. For the eight respondents considering leaving Ireland in the next few years – either to return home or to move to a third country – all but one mentioned the desire to reunite with friends and family as a reason. Some sought to reunite with siblings, others with their adult children whom they had been unable to bring to Ireland with them:

"If my family can come, then I can stay, but as long as my family is there and they can't come over here, no, then I can't think of living alone here for long, no" (Shirley, India, 40 s).

The decision to emigrate to ensure the integrity of the family unit was particularly difficult for those who had moved to Ireland specifically to reunite their families after years of separation while nursing in countries that prohibited family reunification:

"I grabbed the opportunity ... You know, my goal at that time was to bring my families with me. I don't care how much is the pay or you know, as long as I can bring. Because I've been away from my kids for four years ... I decided ... okay I'm going away to a place where I can bring my kids with me. So this is the opportunity that came, that's why I grabbed it" (Carol, Philippines, 40 s).

In addition to those who sought amenable family reunification policies to enable their adult children to continue to reside with them, respondents with young children also spoke of their desire for improved family reunification policies to enable them to bring grandparents to Ireland for periods of time to assist with child care. Given the distances and travel costs involved, it was felt that the current three-month limit on such visits made the arrangements unfeasible. In return for their labour, respondents sought to live in Ireland with their spouses and children and also to maintain contact with other family members "back home" – for instance, by having their own parents or their adult children visit them in Ireland for extended periods.

#### Residency and naturalization

A related concern for respondents (13) was the issue of long-term residency and citizenship, in that Ireland's naturalization procedures effectively meant that the integrity of families currently living together was threatened. Rather than apply for residency/citizenship as a family unit, each member of the household must apply for residency/citizenship separately. Although all family members may eventually achieve the same status in Ireland, in the meantime they hold a variety of immigration stamps and citizenship entitlements:

"It's alright for us because we can apply for long-term residency or citizenship after five years and there will be no problem because we can carry our husbands, our spouses. But then our children is the problem, you know" (Carol, Philippines, 40 s).

Migrant nurses are entitled to apply for citizenship when they have worked in Ireland for five years [29]; however, their dependent spouses must also wait until they have worked in Ireland for five years before they can apply. Given that dependent spouses of non-European Union workers had no right to work prior to 2004 [30], 2009 – one year after Ireland entered an economic recession – is the earliest possible date that nurses' spouses who entered the country as dependents could begin naturalization procedures. Under the current rules, one family member may acquire Irish citizenship while the rest of the family continue to renew their visas and immigration stamps.

This is of concern to respondents, but their main fear is that family members, particularly children, may never achieve residency or citizenship in Ireland. When calculating the five years' residency required to achieve Irish citizenship via naturalization, time spent in full-time education is not considered [31]. Essentially this means that the children of migrant nurses, regardless of how long they had lived in Ireland, reach the end of their second-level education without any entitlement to apply for either long-term residency or citizenship [32] because time spent in the State "for the purposes of study" [31] does not count.

"No, there's no hope, they will apply as an individual and it's more ... difficult for my kids because when they reach the age of 16, they have their own garda card and they have to apply for their own visa" (Agatha, Philippines, 50 s).

A related issue for the children of migrant nurses was that, despite their residence in Ireland and their parents' employment in Ireland, they were not entitled to subsidized university fees, as Irish students are:

"And they're going to college, we have to pay lots of money, you know, seven thousand a year ... we are wondering why there's a difference between us and an Irish [parent] because we are also paying the same tax" (Agatha, Philippines, 50 s).

This prompted a difficult dilemma for migrant nurse parents as their children approached the end of their secondary education and sought to continue into university education. The options were to pay non-European Union university fees for their children to remain with them in Ireland (EUR 15,000+ per annum, but free for Irish and European Union citizens); to send their children back home for their university education; or to attempt to secure an Irish work permit for their children:

"... what's the point of staying here if they're [children] not happy, you know? Especially my daughter wants to study, wants to study computers, but she can't. I don't know if she can work" (Lisa, Philippines, 40 s).

"I had a friend and ... eldest daughter is studying nursing now, I think it's third year now and her second daughter just passed the leaving cert and qualified for nursing, so it's a huge and she can't afford it any more – 13 grand a year for two students" (Regina, Philippines, 40 s).

The other alternative was for respondents to relocate as a family unit to another developed country that will provide long-term residency or naturalization to both migrant nurses and their families, thereby ensuring the long-term integrity of the family unit:

"The kids are not happy that we're going to move in Canada, they're very settled here, they like the place, they like the people" (Monique, Philippines, 30 s).

"Australia is offering, like as soon as you go, you go with your permanent residency. America is offering green card straight away" (Sheela, India, 20 s).

Naturalization procedures were further complicated by the significant delays in processing citizenship applications. At the time of interview, this had led some respondents to question whether they would ever actually acquire Irish citizenship:

"I don't know if I'll be able even to get my Irish passport after 10 years, I don't know, it's very unclear" (Francesca, Philippines, 30 s).

Once again, this prompted international comparisons:

"... You know for example, his sister was in UK, she's been there for five years and now she's a citizen of UK, but that doesn't happen here, you work for five years and you don't become a citizen of Ireland like that" (Sheela, India, 20 s).

Although all respondents had secured permanent employment, some had purchased houses and all seemed to feel generally financiallysecure in Ireland, uncertainty surrounding citizenship entitlements caused respondents to question whether they had a long-term future here:

"Make us stable here, not just financially, but, you know, stability as, as citizens ... We're not here as just to work, we also want a place to live, you know what I'm saying?" (Clara, Philippines, 30 s).

"If they can give me Irish citizenship then I would be very, very happy because now I can make my home" (Ivory, Philippines, 50 s).

These findings correspond to calls by researchers and policy analysts, both in Ireland [29,33] and internationally [4,34] for a more holistic approach to migration, one that ensures that "the wider reality of migrants' lives forms part of the focus of public policy" [29]. In the Irish case, a specific "fast track" visa scheme was developed to facilitate the migration of migrant nurses to Ireland [2]. It was later modified to enable the spouses of migrant workers to obtain employment in Ireland [30] and it would appear that further modification may be necessary to address the issues raised by respondents in relation to residency, naturalization and family integrity.

#### Stability in emigration

The uncertainty and inconsistency evident in Irish migration, residence and naturalization procedures encouraged respondents to examine their options globally. Comparisons between the Irish and Canadian or American models were a common feature of interviews. Respondents noted that in addition to having clear-cut immigration and residency procedures, countries such as Canada also allowed skilled migrants to sponsor children aged over 18 years and other family members, such as parents or siblings, to join them. Thus, in addition to ensuring the integrity of the family unit, migration to Canada was also seen as enabling respondents to offer migration opportunities to other family members:

"I'm already satisfied ... I'm happy with my job and I just want to go to Canada for my daughter, so that she can have a chance" (Vina, Philippines, 40 s).

For those whose spouses were currently unemployed or underemployed (four) in Ireland, migration was seen as an opportunity for career advancement or the opportunity to use their professional qualifications.

"Well, my husband really would like to go to America, probably that's only the reason. If you asked me, honestly speaking, I'm sort of settled and happy now here in Ireland, I wouldn't go anywhere else" (Francesca, Philippines, 30 s).

#### Opportunities overseas or retiring back home

Onward migration also offered professional opportunities. For instance, one respondent spoke of her delight at being offered the opportunity to work in her area of expertise in Canada, something denied her in Ireland. She also spoke of the generous relocation package offered by a Canadian employer to facilitate her relocation. Another spoke of the lower cost of living in Canada. Others spoke about keeping their options open in terms of emigration:

"Maybe for as long as I'm nursing, I'll be staying here in Ireland, but, em, I don't know, because at the back of my head, I still have the notion of going to America, that's to be honest" (Fatima, Philippines, 30 s).

Six respondents who planned to work in Ireland until retirement and then move back to their home countries following their retirement were certain of their plans:

"Two years more in Ireland then I will retire because I think I need to retire. I said, I need to enjoy, not always working. I been working since I was 19, I was already a nurse and now I'm already 51 so I said, I'd like to go home that I'm still able" (Ivory, Philippines, 50 s).

"The minute I retire, I'm going home straight to Africa" (Paddy, Nigeria, 40 s).

Those respondents who had definite plans to return home appeared among the most satisfied in Ireland. Perhaps that is because they had low or minimal expectations of Ireland beyond a continued right to work and earn a salary. Their version of migration – to stay, earn and leave – was perhaps also a closer "fit" with the Irish model of migration.

#### Mixed feelings about leaving

The 11 respondents who were planning to leave Ireland, including the three with immediate plans, had mixed feelings about doing so. Although excited about the opportunities available to them elsewhere, their excitement was also tinged with regret at having to leave Ireland,

"But I'm telling you, if I'm going to leave this place, this Ireland ... really had a spot in my heart and I say, Oh my God, I'll be crying, you know because I'm really already settled" (Monique, Philippines, 30 s).

Even some of those with definite migration plans had previously planned to remain in Ireland for the medium to long term, as evidenced in the long-term investments, both financial and social, that they had made in the country. Respondents appeared to have been unaware until recently of the difficulties that would force their emigration. In this case, the realization dawned as the children of this respondent approached school-leaving age:

"Because when we got a house last year, we were really, a hundred percent decided to stay here, you see. But when we know about the laws that's, I said 'oh God"' (Carol, Philippines, 40 s).

There was a sense of frustration among respondents at having to leave Ireland, having already invested considerable time and energy in settling here:

"We work hard, we sacrifice a lot and then we cannot still stay. I don't know, it's so difficult to accept, but that's the way it goes" (Clara, Philippines, 30 s).

"We just want, really, a place to live in. Because it's hard to start and start and start again, you know' (Clara, Philippines, 30 s).

Frustration stemmed from the fact that there was still a nursing shortage in Ireland, but that the procedures that might enable them to remain – in terms of naturalisation, residency or immigration – were not in place. On an individual or a family level, respondents felt they could not afford to wait in Ireland in the hope that these issues would be resolved. Despite expressions of regret about the possibility of onward migration, respondents had no hesitation in explaining that their primary concern was their families:

"I'm sorry, but I'm not going to stay in Ireland. I love to stay here because it's quiet, it's a safe place, it's a good thing, you know, those things, but the only, we need our family, you know, that's the most, I think that's the number one, family" (Ivory, Philippines, 50 s).

Respondent nurses had been involved in the international recruitment "game" for some time and were acutely aware of the need to look after their own interests and maintain the integrity of their family unit, whatever the cost.

## Discussion

### Onward migration

The main finding to emerge from interviews with migrant nurses was that over half (11) of those interviewed intended to leave the country within the next five years. Verification figures from the Irish Nursing Board would appear to support this finding. In 2008, 1885 Indian and 261 Filipino nurses sought to have their Irish registrations verified by nursing boards in countries such as Australia, Canada and the United Kingdom (Irish Nursing Board, unpublished data) [14]. In other words, they had expressed their intention to migrate from Ireland. This could be seen to confirm the conclusion that "migration is becoming increasingly transitory"[35] or to support the belief that international recruits are unreliable [4]. However, most nurses interviewed in this study were leaving because Ireland had failed to provide sufficient long-term security, via residency or citizenship entitlements, to their families.

Although we had anticipated that the onward migration of migrant nurses might be an issue to emerge from the research, we had expected workplace-related factors or salary and cost-of-living issues to play a deciding role in the decision: in other words, factors endogenous to the health system [28]. Instead, interviews revealed that the desire to be settled and stable was more important for respondents and far outweighed career-related considerations and other endogenous health systems factors in determining whether they would stay or leave Ireland.

Those who planned to remain spoke of being settled. Those who planned to leave Ireland regularly cited the desire for stability as a major consideration. They were considering or already planning to migrate to countries with more progressive immigration/citizenship regimes in which the acquisition of residence or citizenship for themselves and for their families was more straightforward and deemed to have a better chance of success.

Whereas respondent nurses prioritized stability, destination countries such as Ireland hold a more short-term view of migration and are less likely to automatically provide migrants with entitlements to permanently settle. These "dramatic shifts in the destinations of migration, restrictions on residency and strict limitations on settlement" [36] have fundamentally altered migration for individual migrants, and for source and destination countries.

In the face of these changes, traditional explanations of migration, which emphasize the movement of people "pushed" from the source country and "pulled" towards the destination country so as to improve their financial situation, provide a limited and even misleading framework as the "rationale of economic calculation that this model presupposes is also too limited to embrace the complex motivations of migrants" [36]. This explanation of migration is over-simplistic when compared with the complex range of factors considered by migrant nurses in making their migration decisions (see Table 1) and fails to take into consideration the fact that, far from involving a single permanent move [37], migration movements today "are increasingly sequential, involving more than one destination" [38]. Respondent decisions to remain in or to leave Ireland involved weighing up a complex range of factors, such as considering children's future educational needs and perhaps the long-term care needs of elderly parents, in addition to any personal or financial motivations for migration. As Papastergiadis explains:

**Table 1:** Factors influencing migrant nurse decisions to stay or leave Ireland (adapted from Padarath, 2004 [28])

	Endogenous (within the health system)	Exogenous (outside the health system)
Push (from Ireland)	De-skilling (brain waste)	• Uncertain residency/citizenship entitlements (especially for children) • No family reunification rights for extended family (children over 18, parents) • No entitlement to subsidized third-level education for children • No protection for the integrity of the family unit

Pull (to Canada/USA/Australia)	• Relocation bonuses • Opportunity to specialize and/or use one's specialty • Opportunity to advance career	• Clearer whole-family residency and citizenship entitlements • Opportunity for family reunification • Integrity of family unit protected

Stay (in Ireland)	• Job security (permanence) • Salary enabling remittances • Maternity leave entitlements • Equality	• Desire to be settled • Avoid further disruption (for children) • Feel safe • Equality

"The constraints of the past and the possibilities of the future are carefully weighed in every decision to migrate. From such a perspective the question of personal choice may simply seem like the wrong question. It gives too much attention to the individual's present action, and blurs the complex networks of responsibilities that link a person to the past and future" [36].

Table 1 is an adaptation of Padarath's "push-pull, stay-stick" model of health worker migration [28], which takes into account the experiences of migrant nurses in Ireland as they consider whether to stay or re-emigrate to other destination countries. It reveals that exogenous factors, i.e. factors outside the health system, were most influential in the decision-making process when migrant nurses were considering re-migration. This should serve as a "wake up call" for health service employers and health workforce planners, as it undermines several commonly held assumptions about the migrant nurse workforce.

The first unfounded assumption is that migrant nurses from the developing world will be grateful to obtain employment in destination countries, such as Ireland, and will remain here for as long as required. An Irish Director of Nursing involved in the early recruitment campaigns claimed to have been "greeted as 'a god' when she was in the Philippines to interview applicants" [39]. She proceeded to explain that migrant nurses were merely a short-term solution to the nursing shortage and that, as they become available, Irish nurses "will have first priority for jobs" [39].

This presumption, held by Irish employers including those involved in international nurse recruitment, "that migrant workers are essentially available on tap" [29] is a dangerous one, as it lulls workforce planners into a false sense of security, assuming that any skills shortfalls nationally can be met from a global skills pool, presumed to be unlimited. It also presumes that migrant nurses have a limited set of options in terms of migration, which is far from being the case. Secondly, the findings disprove the assumption that migrant nurses tend to be young, single and motivated primarily by financial gain. The primary objective of migrant nurse respondents – regardless of age or marital status – was to achieve stability for themselves and their families, specifically for their children and for their parents.

Thirdly and finally, the research findings reveal as unfounded the impression that health employers or even the health system acting alone can apply strategies to retain migrant nurses in post, for example via the provision of permanent posts or via general retention measures. In 2002, Buchan noted that nursing shortages were a health systems problem requiring health systems solutions [5]. Our findings suggest that many of the solutions, at least in Ireland, lie outside the scope of the health system and require a wider policy response from government departments with responsibility for migration, family reunification, naturalization and education. Sustaining Ireland's reliance on internationally recruited nurses in the medium to long term will necessitate a much better understanding of the dynamics of nurse migration and a far more coherent approach to migration, involving joined-up policy thinking between various government departments and agencies.

### Recruiting nurses or nursing units?

Like most destination countries, Ireland appears to have envisaged international recruitment campaigns as a means of importing hard-working nurses on a temporary basis as a stop-gap solution to staffing shortages in the health system [39]. The underlying aim seems to have been to import nursing "units" with minimal regard for the individual nurses beyond arrival and adaptation. However, the reality is that migrant nurses are individuals who seek what many of us take for granted – a job, a salary, a family life. For some, Ireland may be just the latest in a long line of destination countries that have failed them in their quest for a home (away from home) in which they can settle with their families as well as work.

The contradiction at the heart of the matter is that, despite the recognized need for migrant nurses, migrants are generally afforded a much cooler reception by destination countries [8]. This ambiguity is played out in the everyday experiences of migrant nurses in Ireland who, although actively recruited internationally, find their longer-term settlement and integration and that of their family, impeded by migration policies designed to accommodate lone workers migrating to work on a temporary basis, a system ill-equipped for the long-term retention and integration of health workers and accompanying family members. Changes to the migration system, designed to retain migrant nurses in Ireland, have been instigated to avoid a "possible negative impact to both our healthcare services and private industry" [30], rather than to improve the quality of life of individual migrant nurses.

This highlights Ireland's relative inexperience as an immigration destination, but it also reveals underlying assumptions about the nature of migration, specifically nurse migration. Migration policy continues to treat migrant nurses as a short-term, renewable resource, as "disposable cogs on a global assembly line of caregivers" [4]. The presumed abundance of migrant nurses internationally mitigates the need to retain them in service.

Although destination countries, such as Ireland, compete fiercely to recruit migrant nurses from their countries of origin and subsequently to recruit them from other destination countries – Filipino nurses are recruited from the Philippines to Saudi Arabia, recruited onwards to Ireland and then to Canada or Australia – the effort put into retaining them is negligible [16]. The health workforce has been transformed by globalization and migration and yet the fundamental challenge – to retain nurses in the health system – remains unchanged.

### Long-term need versus temporary migration

Ireland's migration policies to date have focused on filling the specific skills needs of the economy via migration, for instance by actively recruiting migrant nurses to fill vacancies in the health services. But this approach, which sees migrants only as workers, is unsuited to the long-term retention of migrants and their families:

"Failure to recognise the strength and importance of family ties and to consider a broader approach, may force many migrants currently living in Ireland, or future potential migrants, to consider other countries with more favourable and clearer family reunification policies as their preferred work-destination" [33].

"Migrants come not simply as labour units, useful for a while, but ultimately dispensable ... Insofar as migrants and their families may come to Ireland and for as long as they remain in Ireland, it is important that the wider reality of migrants' lives forms part of the focus of public policy" [29].

The suggestion is that a failure to attend to wider integration issues, such as family reunification, residency and citizenship entitlements, will result in a failure to attract or retain skilled workers such as nurses for whom there continues to be a demand. The short-sighted, economically-driven model of migration currently in place in Ireland has much in common with the "*Gastarbeiter*" (guest worker) migration systems favoured in mainland Europe in the 1970s:

"So far as the economy of the metropolitan country is concerned, migrant workers are immortal: immortal because continually interchangeable. They are not born: they are not brought up: they do not age: they do not get tired: they do not die. They have a single function – to work. All other functions of their lives are the responsibility of the country they came from" [40].

Given that the very functioning of the health system relies upon migrant nurses, the fact that some of Ireland's migrant nurses have expressed a desire to settle here should be seen as an opportunity. However, as our findings have shown, in order to retain these nurses in the health system on a long-term basis, it will be "necessary to address the nurses' migration experience as a whole" [34]. The current contradictions between Irish health and migration policies, whereby the migration system militates against the long-term settlement of migrant nurses and their families, may prove detrimental to a health system heavily reliant on a migrant workforce. In the meantime, actively encouraging nurses to work in Ireland – without putting in place policies and procedures to enable them to settle here with their families – indicates weak policy-making capacity by the Irish Government. It also signals a reluctance to extend to migrants the protections afforded the family within the Irish Constitution [33].

### Improving workforce planning

"Whatever the circumstances, an effective workforce strategy has to focus on three core challenges: improving recruitment, helping the existing workforce to perform better, and slowing the rate at which workers leave the health workforce" [41].

Measuring the performance of health systems in addressing these three challenges requires evaluative evidence. Poor data availability is a frequent problem for health systems internationally; most require "much more detailed data collection, careful planning, and evaluation of the health care workforce" [4].

In the Irish context, an understanding of the dynamics of nurse migration and its impact on the nursing workforce is hampered by a lack of data [2]. Hongoro and Normand (2006) highlight the importance of human resource models in enabling health planners to estimate the length of a nursing career and to plan accordingly [42]. Without even a basic profile of its migrant nurse workforce, it is difficult to see how migrant nurses can be properly incorporated into Irish workforce planning strategies, or how their retention might be measured, let alone improved. Improved data are necessary to enable Ireland to incorporate nurse migration into "the overall workforce planning approach" [43] and in order to move away from the perception of international nurse recruitment "as a cheap option with 'expendable' migrant health professionals" [43].

## Conclusion

In many cases, Ireland is the latest in a long line of destination countries to have failed respondents in their quest for a home in addition to an overseas nursing post. The findings illustrate the sacrifices behind the global migration of nurses and serve as a timely reminder – to policy-makers in Ireland and globally – of the social costs of nurse migration [4]. Countries, like Ireland, that rely heavily upon migrant nurses should not become complacent and presume that successful international recruitment campaigns have permanently "solved" their nursing shortages. International recruitment may defer but will not resolve nursing shortages unless implemented alongside retention measures to keep nurses (both local and migrant) in post.

What is required is a more holistic and system-wide approach to international nurse recruitment, which recognizes that migrant nurses must adapt to life outside the health system as well as to Irish nursing practice and that the acquisition of a permanent nursing post means little if not accompanied by long-term residency and citizenship rights for migrant nurses and their families. This will necessitate a sea-change in Irish migration policy, which to date has been market-led. What is required is a policy that recognizes the contribution migrants make – both in terms of skills and in terms of social contribution – and that encourages their long-term settlement and integration into Irish society. However, just as international recruitment initially offered destination countries a "quick fix" to nursing shortages, there may be little incentive to resolve underlying problems until the pool of international nursing recruits begins to dry up.

## Competing interests

The authors declare that they have no competing interests.

## Authors' contributions

NH carried out the interviews and data analysis and drafted the paper. RB and HMG designed the study and provided editorial comment on the draft paper. All authors have read and approved the final manuscript.
